# Metal-organic frameworks and plastic: an emerging synergic partnership

**DOI:** 10.1080/14686996.2023.2189890

**Published:** 2023-03-28

**Authors:** Teresa F. Mastropietro

**Affiliations:** Department of Chemistry & Chemical Technologies, University of Calabria, Rende, Italy

**Keywords:** Nanoplastics, MOFs, adsorption, catalytic degradation, membranes, PET reuse

## Abstract

Mismanagement of plastic waste results in its ubiquitous presence in the environment. Despite being durable and persistent materials, plastics are reduced by weathering phenomena into debris with a particle size down to nanometers. The fate and ecotoxicological effects of these solid micropollutants are not fully understood yet, but they are raising increasing concerns for the environment and people’s health. Even if different current technologies have the potential to remove plastic particles, the efficiency of these processes is modest, especially for nanoparticles. Metal-organic frameworks (MOFs) are crystalline nano-porous materials with unique properties, have unique properties, such as strong coordination bonds, large and robustus porous structures, high accessible surface areas and adsorption capacity, which make them suitable adsorbent materials for micropollutants. This review examines the preliminary results reported in literature indicating that MOFs are promising adsorbents for the removal of plastic particles from water, especially when MOFs are integrated in porous composite materials or membranes, where they are able to assure high removal efficiency, superior water flux and antifouling properties, even in the presence of other dissolved co-pollutants. Moreover, a recent trend for the alternative preparation of MOFs starting from plastic waste, especially polyethylene terephthalate, as a sustainable source of organic linkers is also reviewed, as it represents a promising route for mitigating the impact of the costs deriving from the widescale MOFs production and application. This connubial between MOFs and plastic has the potential to contribute at implementing a more effective waste management and the circular economy principles in the polymer life cycle.

## Graphical abstract

**Figure:**
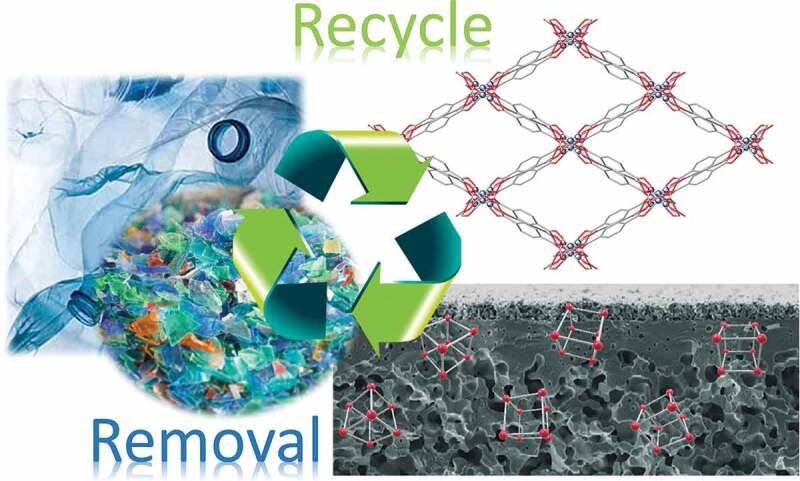

## 1. Introduction

**Figure 1.**
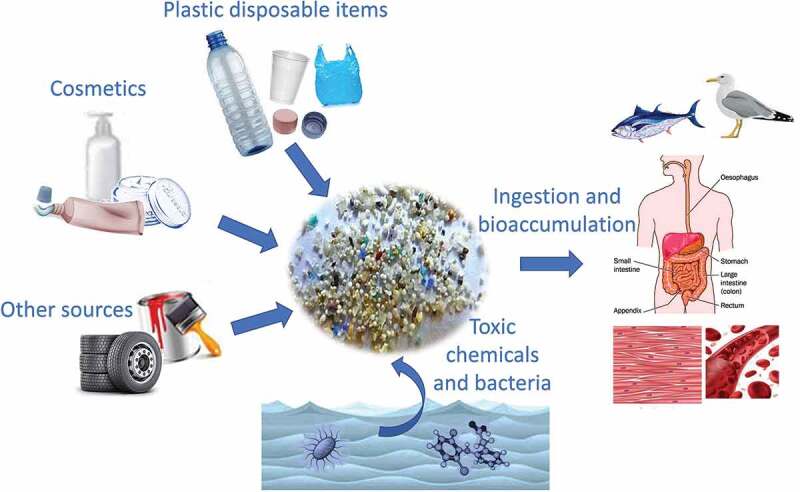
Major sources and fate of MPs and NPs, as indicated in: https://echa.europa.eu/hot-topics/microplastics.

Plastic pollution has become one of the most urgent environmental issues of the 21st century. The exponential increasing manufacture of disposable plastic goods has overwhelmed the world’s ability to handle them properly. The incorrect and ignorant disposal of plastic materials, especially those meant for single use, has rapidly led to uncontrolled plastic accumulation in the environment. This ubiquitous and persistent pollutant afflicts lands, watercourses, and oceans [1,2], creating problems for wildlife and their habitats as well as for human populations. Once in the environment, the continuous exposure to the activity of sunlight, wind, and ocean waves breaks down plastic debris into smaller particles, the so-called microplastics (MPs, 0.1 μm to 5 mm in size) and nanoplastics (NPs, 1 nm to 100 nm size) [3,4]. MPs/NPs can also be produced by mechanical abrasion during manufacturing and transformation processes and are often intentionally added to a wide range of different daily use products (Figure 1) [5]. MPs/NPs can easily spread in every corner of the globe and represent the most challenging type of micropollutants, which are nearly impossible to recover [6–9]. MPs/NPs have been found in municipal drinking water systems [10], in food [11], and in tissues and blood stream of aquatic living organisms, animals, and humans [12–14].

MPs/NPs can feature different physical structures, such as fibres, beads, sheets, or irregular fragments with a polydisperse size distribution. They can have a porous structure and can form aggregates or colloidal suspensions, depending on their size and density [4]. From a chemical point of view, MPs/NPs are made of organic polymer chains, which can be linear or branched, with very different structures and chemical composition (Table 1), including polyethylene terephthalate (PET), polyethylene (PE), polypropylene (PP), polyvinyl chloride (PVC), poly(lactic acid) (PLA), polyamide (PA), polystyrene (PS), polyurethane (PU), and polycarbonates (PC), often mixed with functional additives, including organic dyes [5,15]. Although MPs/NPs are assumed to be relatively inert, they can adsorb dissolved harmful contaminants, such as heavy metals [16], organic chemicals [17,18], or pharmaceutical compounds [19], as well as pathogens [20] onto their surface, aggravating their ecotoxicological effects [21] and further increasing risks for human and other living species’ health. MPs/NPs are inherently neutral; but most of them feature a net and generally inhomogeneous surface charge due to both the adsorption of co-existing contaminants and weathering processes [4,22]. The MPs/NPs particle size mainly determines their sorption capacity and also their environmental fate [23,24]. Since NPs have larger surface area than MPs, and more accessible binding sites, they typically display higher sorption rates than MPs.

**Table 1.** Main MPs/NPs chemical composition and structures.

Entry	Polymer	Structure	Main characteristic
**1**	Poly(ethylene terephthalate) (PET)	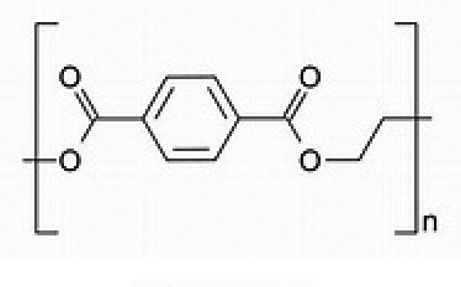	Thermoplastic polymer Semicrystalline
**2**	High density polyethylene (HDPE)	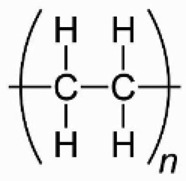	Thermoplastic polymer Flexible
**3**	Low density polyethylene (LDPE)	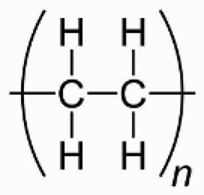	Thermoplastic polymer Branched on ~2% of the carbon atoms Semirigid
**4**	Polypropylene (PP)	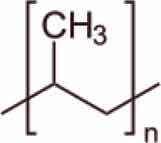	Thermoplastic polymer Homopolymer or ethylene copolymer Basic chain structures: Atactic (aPP) – Irregular -CH_3_ arrangement Isotactic (iPP) – -CH_3_ arranged on one side of the carbon chain Syndiotactic (sPP) – Alternating -CH_3_
**5**	Polystyrene (PS)	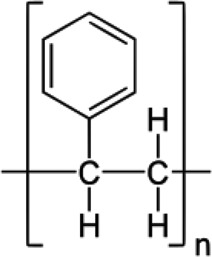	Thermoplastic polymer Basic chain structures: Atactic (Ps-at) – Irregular -Ph arrangement Isotactic (PS-it) – Ph arranged on one side of the carbon chain Syndiotactic (PS-st) – Alternating -Ph
**6**	Polyvinyl chloride (PVC)	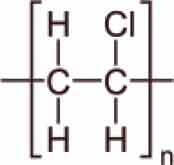	Thermoplastic polymer Basic chain structures: Atactic (PVC-at) – Irregular -Cl arrangement Isotactic (PVC-it) – Cl arranged on one side of the carbon chain Syndiotactic (PVC-st) – Alternating -Cl
**7**	Poly(lactic acid) PLA	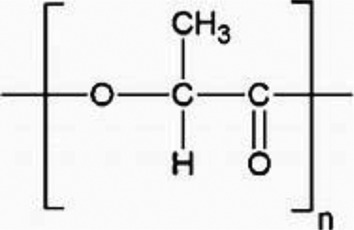	Thermoplastic polymer Amorphous glassy to semi-crystalline
**8**	Polyvinylidene fluoride (PVDF)	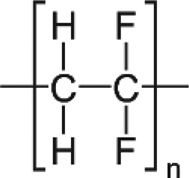	Thermoplastic polymer 50–60% crystalline Piezoelectric
**9**	Poly(methyl methacrylate) (PMMA)	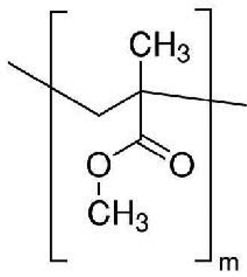	Thermoplastic polymer Amorphous Lightweight or shatter-resistant
**10**	Others: for example, i) Polyurethanes (PU) ii) Polycarbonate (PC) iii)Polyamide (PA)		i) A class of polymers with repeating units linked by carbamate (−NH−(C=O)−O−) bonds ii) A class of thermoplastic polymers containing ((C=O)−O−) groups iii) A class of polymer with repeating units linked by amide (−NH−(C=O)−) bonds

In recent years, due to the increased awareness and worldwide concern, many countries have approved laws and undertaken actions to ban several plastic products [25–27], with the aim to reduce the source of MPs/NPs, but the elimination of MPs/NPs from the environment will requires a long time period and the efforts of several generations. Several methods conventionally used for wastewater treatment have been applied for MPs/NPs removal and have been widely reviewed [28–31], membrane separation being one of the most promising techniques [32–34]. Although the current technologies have the potential to remove MPs/NPs, many of these processes are not suitable, being intrinsically complex and requiring high maintenance costs and energy consumption [35]. Moreover, the removal efficiency for NPs is generally low by using a single treatment and integrated and combined removal technologies are instead required [36]. Therefore, there is a pressing need to consider novel non-conventional strategies for efficiently removing MPs/NPs from water.

Metal-organic framework (MOFs), also called porous coordination polymers (PCPs), are a class of relatively new crystalline nano-porous materials, generated by the coordination of bidentate or multidentate organic ligands to metal ion cores or metal clusters [37–39]. Generally, MOFs have unique properties, such as strong coordination bonds, large and robustus porous structures and accessible surface areas, and high adsorption capacity, which make them suitable adsorbent materials for wastewater treatment, reaching high performances in particular for the removal of micropollutants [40–43]. All these features, along with the enhanced catalytic activity that they can display [44–46], provide an exciting opportunity to produce new advanced adsorbents for MPs/NPs removal from water and wastewater [47] as well as catalytic materials able to easily degrade MPs/NPs [48]. Moreover, MOFs and MOF-based materials, including both composite materials and innovative MOF-modified membrane-based filtering systems, offer a chance for the simultaneous removal of MPs/NPs and other coexisting micropollutants with a single process. Indeed, MPs/NPs pollution typically occur in complex matrices [49,50]. Additionally, a pool of dissolved organic carbon (DOC) can be generated from MPs/NPs by ultraviolet irradiation from sunlight exposure, which activate the photolytic cleavage of the polymer chains, provoking the release of shorter fragments, photo-oxidized products, monomers, and additives in water [51–53]. All this low molecular weight contaminants could be captured by MOFs by adsorption within their porous network [54,55]. Despite these promising perspectives, however, the literature on this topic is limited. This review provides a survey of the emerging findings on the use of MOFs for MPs/NPs removal and degradation. A schematic summary of the applications of MOFs and MOF-based composite materials for adsorption and degradation of MPs/NPs is reported in Table 2.

**Table 2.** Applications of MOFs and MOF-based composite materials for adsorption and degradation of MPs/NPs.

Entry	MOF or MOF-based composite	MPs/NPs	Application	Conditions	Process efficiency	Ref.
**1**	ZIF-67	PS (1–3 µm)	Adsorbent	5 ppb of MPs in water at a 3–12 pH range, 288–308 K	Up to 92.1% of removal at 298 K	[56]
**2**	MIL-101(Cr)	PS (65 nm)	Adsorbent	5–100 ppm of NPs in MiliQ water at pH 5–10, 25°C	Up to 96% of removal at 5 and 70 ppm, pH 5	[57]
**3**	UiO-66-X in a melamine porous foam, X = H, NH_2_, OH, Br and NO_2_	PVDF 260–322 nm PMMA 325 nm PS 183 nm	Adsorbent	0.5–2 ppm of MPs in water/ethanol (3:1) or simulated seawater environments	PMMA, 88.2% PS 85.7% PVDF Up to 95.5% of removal	[58]
**4**	ZIF-8 in a wood aerogel	PVDF (60—110 nm) PS (90―140 nm)	Adsorbent	0.5 ppm of MPs in water/ethanol (3:1) and seawater environments	Up to 91.4% of removal	[59]
**5**	PSF/MIL-100 (Fe) mixed matrix membrane	PVC (134.6 µm) PE (42.5 µm)	Adsorbent	Different concentrations of MPs/Nps in water at different pHand in presence of MB	Up to 99% of removal	[60]
**6**	UiO-66(Zr)	PET chips	Catalytic degradation	Solvent-free process 5 mol % of UiO-66(Zr) 24 hours at 260°C under 1 atm of H_2_ or Ar in presence of PP or PE	Up to 98% of degradation	[48]

Furthermore, taking into account the growing request for the scale-up of MOFs production at the industrial scale and the urgent need of more convenient preparative routes, dictated by both the shortage of organic and inorganic precursors, and by the environmental impact and the associated costs of MOFs manufacture [61–64], a recent trend for the alternative preparation of MOFs starting from plastic waste is also discussed. Indeed, the use of recyclable waste as a supply for both organic and inorganic constituents offer a promising practice for the sustainable and low-cost production of high-value materials, including MOFs and MOF-composites [65], meeting at once the concepts of circular economy [66,67]. Recently, various significant studies of waste-derived MOFs and MOFs composites have been reported and reviewed [68–71]. This review mainly focuses on the use of PET wastes as a valuable source of ligands for MOFs production, highlighting that the PET-derived MOFs feature similar properties and adsorption performances when compared to MOFs produced with commercial organic ligands.

Finally, challenges and future perspectives are examined, with the aim of promoting the beneficial connubial between MOFs and plastic, encouraging a wider use of MOF-based materials for MPs/NPs removal and stimulating the recycling of plastic waste as an alternative source of MOFs precursors, thus contributing to control both pollution and costs for MOF manufacture.

## 2. MOFs as MPs/NPs adsorbents

Adsorption can be regarded as a feasible technique for MPs/NPs removal from water. It is eco-friendly and cost-effective, since working conditions are generally mild, it requires low energy consumption, and does not produce by-products. Carbon-based materials and layered double hydroxides have been investigated as possible adsorbents for NPs from water [72,73]. Nevertheless, they do not possess specific functionalities toward different kinds of NPs and exhibit low adsorption capacity.

Owing to their high active surface area and porosity, surface charge, tuneable particle size, and functional groups, MOFs can potentially adsorb MPs/NPs and other eventual co-pollutants. Both MPs and NPs size are considerably larger than MOF pore size, consequently, it is unlikely that these particles can be adsorbed within the MOF porous network. Nevertheless, multiple interactions of different nature (electrostatic interactions, hydrogen bonding, halogen bonding or stacking interactions, and van der Waals interactions) can occur between the outer MOF surface, rich of functional groups, and MPs/NPs [47]. Moreover, being MOFs extremely flexible materials, surface functionality can be easily engineered and custom-designed to maximize these interactions, also depending on MPs and NPs composition, as well as to target additional soluble analytes. With this respect, MOFs offer the possibility to remove at once solid MPs/NPs and additional soluble pollutants, that generally occur in complex matrices. Additionally, if MOFs display any catalytic activity toward the plastic polymers, the degradation and adsorption of the resulting products can be accomplished at once.

In a recent research, ZIF-67 was investigated as a viable adsorbent material for MPs from water media [56]. PS MPs with particle dimensions falling in the range of 1.0–3.0 µm were used for simulating MPs waste and their interactions with ZIF-67 particles were studied at a 3–10 pH range and at different temperatures (288–308 K). The authors hypothesized that ZIF-67 can adsorb MPs from water through a combination of different interactions, including hydrogen bonds, electrostatic interactions and π-π stacking. A maximum loading of 11.6 mg/g was reached.

Into another very recent study, published during the writing of this review, MIL-101(Cr) was studied as a potential adsorbent for the removal of PS NPs from MiliQ water [57] at different pH. The mean PS NPs particle size was 65 nm. The best performances in terms of removal efficiency were achieved at pH 5. By using two different initial concentrations of NPs, namely 5 and 70 ppm, a 96% removal efficiency was reached, with a maximum loading of 800 mg/g at 90 ppm. The removal mechanism was mainly governed by attractive electrostatic interactions occurring between the negatively charged surface of PS NPs and positively charged MOF.

Although these recent studies preliminarily demonstrated that MOFs feature useful properties which can be advantageously exploited for MPs/NPs removal, it is also evident that this strategy implies some issues directly linked to the intrinsic nature of MOFs nanoparticles. Indeed, MOFs are conventionally synthesized as nanocrystalline powder, and their direct use in this form implies some drawbacks, such as difficulties to handle MOF samples, suspension instability and agglomeration, accidental release of MOF nanoparticles, difficulties to recover MOF powders, etc., which hamper their practical applications. Shaping MOFs to prepare bulk materials is an effective strategy to facilitate their wider use [74,75].

### 2.1. MOF-based composite materials

The development of MOF-based porous composite materials is of great importance for applications in separation and heterogeneous catalysis for water treatment purposes, since they allow an easy MOFs use and processing. Moreover, MOF-based composite materials can conceivably display improved performances compared to those of the individual components.

**Figure 2.**
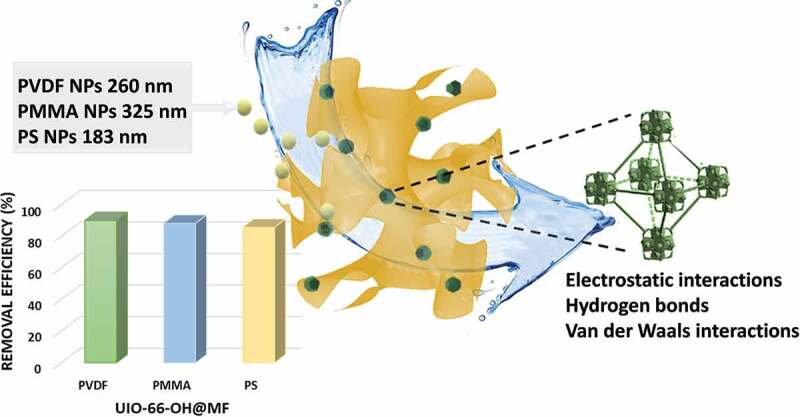
UiO-66-OH@MF removed PVDF, PMMA and PS MPs by filtration/adsorption. Adapted from Ref. [73].

The first proof-of-concept of MOF-based composite materials for the removal of MPs/NPs was reported by Chen et al. in 2020 [58]. A melamine porous foam (MF), a soft material endowed with elevated robustness and stability, was chosen as support for a series of zirconium-based MOFs (UiO-66-X, where X = H, NH_2_, OH, Br and NO_2_), see Figure 2. The MOF loading was varied in the range of 4.4–25.8 wt%. The composite materials were applied in the filtration of various suspensions containing polyvinylidene fluoride (PVDF), poly(methyl-methacrylate) (PMMA) and PS MPs (mean size in the range 180–322 nm) at different concentrations (0.5 to 2.0 g L-1) in water/ethanol mixture (3:1) or simulated seawater environments. The best performing composite material, indicated as UiO-66-OH@MF with a MOF loading of 25.4 wt%, efficiently removed PVDF MPs with a removal efficiency up to 95.5%, maintaining elevated performances during several filtration cycles. The authors hypothesized that MOF nanoparticles decorated with several functional groups or having a high number of defects can adsorb MPs on their outer surface by a combination of weak interactions, like hydrogen bonds, electrostatic and Van der Waals interactions.

A zinc MOF composite, ZIF-8@Aerogel, was reported by You et al. in 2021 [59]. This material was prepared by growing *in situ* ZIF-8 particles on wood aerogel fibres and it was effectively applied for the removal of MPs/NPs at different concentration in water and seawater media. Poly(1,1- difluoroethylene) (PVDF, particle size 60—110 nm) and PS (particle size 90―140 nm) were used to simulate MPs/NPs suspensions. The removal efficiencies of MPs/NPs reached 91.4% and 85.8% in water, for poly(1,1- difluoroethylene) and PS, respectively, and maintained high performances in filtration tests of large-scale seawater MPs/NPs suspensions. The main mechanism of adsorption was supposed to be mainly electrostatic in nature, established between the negatively charged surface of MPs/NPs and the positively charged ZIF-8 particles, although further supported by hydrogen bonding interactions.

### 2.2. MOF-based membranes

Pressure-driven membrane processes are well-established techniques for water wastewater treatment. They ensure high selectivity, reduced energy utilization, possibility to design a continuous-flow mode operation and to shape modular, compact, and easy scalable systems [76]. Standard membranes used for water and wastewater treatment are typically made up of synthetic organic polymers, properly selected and designed to reach the highest performances in different water media, depending on the characteristics of both the solution, including the presence of dissolved salts, pH value, and temperature, and the solute, i.e. chemical composition, presence of functional groups, particle size, and hydrophobicity [77–79]. Three main mechanisms are at the basis of membrane separation: sieving due to size exclusion, repulsive electrostatic interactions (between eventually charged dissolved species and the membrane surface), and adsorption. Different kinds of membranes have been employed for the removal of MPs/NPs from water and wastewater, ultrafiltration membranes showing the better performances [80,81]. However, polymeric membranes feature some disadvantages, such as low hydrophilicity and anti-fouling properties, limited mechanical stability and chemical inertness, which considerably hamper their use in widescale applications [82,83]. Moreover, the concentration of MPs/NPs, especially when NPs are concerned, can deeply influence the performance of conventional membrane filtration processes, particularly nano- and ultrafiltration. Indeed, particle accumulation and caking can lead to pore fouling or can ruin or perforate the membrane surface, thus depleting its filtering ability both in terms of *trans*membrane flux and retention ability [84]. Membrane modification through the incorporation of novel nanomaterials within the polymeric matrices has been proposed as a superior technique to overcome these limitations [85–87]. Developing membranes using MOFs as non-innocent fillers represents a promising platform for the effective removal of MPs/NPs and their co-pollutants that remains largely unexplored. MOFs can assure better performance in long-term treatments, by inducing higher hydrophilicity and antifouling properties in the membrane, and determining enhanced water flux, since they offer additional transport pathways for water permeation [88–91].

**Figure 3.**
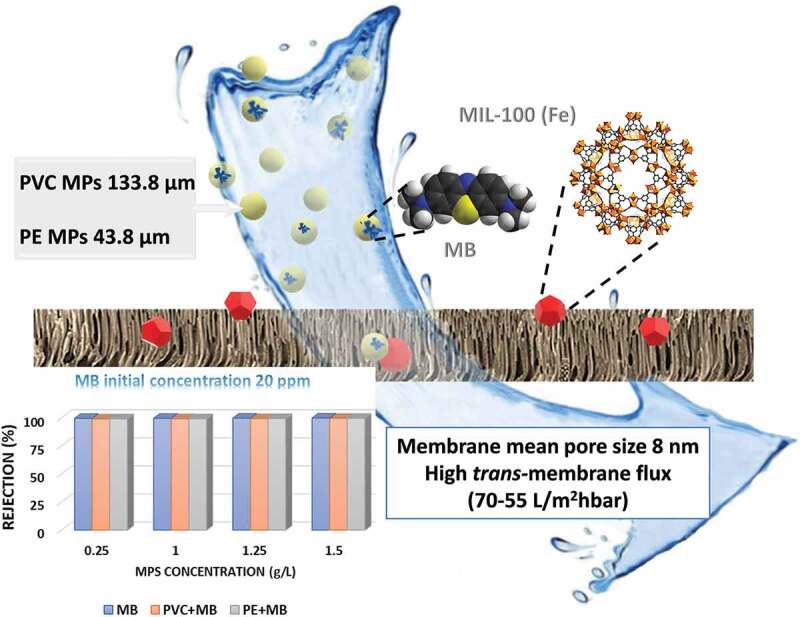
PSF/MIL-100 (Fe) composite membrane removed PVC and PE MPs by filtration/adsorption in presence of methylene blue (MB) dye as co-pollutant. Adapted from Ref. [91].

Only an example of a nanofiltration membrane incorporating MOFs for MPs removal from water has been reported to date, while there are no examples on MOF-based membranes applied for NPs removal. The mixed matrix membrane, indicated as PSF/MIL-100 (Fe), was produced by incorporating MIL-100 (Fe) nanoparticles at different loading (0, 0.25, 0.5, and 1 wt%) into a polysulfone matrix (PSF) [60]. The authors proved that the incorporation of the hydrophilic MOFs at 0.5 wt% loading considerably modified the membrane properties, in particular its morphology and hydrophilicity. The composite membrane demonstrated outstanding performance for several filtration cycles at different concentrations of PVC and PE MPs (0.5, 1, 1.25 and 1.5 g/L) in the presence of methylene blue (MB) dye as co-pollutant (MB concentration range of 10–100 ppm) (Figure 3). The mean MPs size was 42.5 µm for PE and 134.6 µm for PVC. The better performances (rejection values greater than 99% for MB and MPs adsorbing MB) were obtained for pH values in the range of 9–11. In this case, the authors suggest that MB is likely adsorbed on the surface of MPs through electrostatic interactions, and the mixed matrix PSF/MIL-100 (Fe) membrane successively retains both MPs and MPs adsorbing MB through a synergic combination of size-exclusion mechanisms and repulsive electrostatic interactions. The membrane also featured improved water flux and antifouling properties.

## 3. MOFs for MPs/NPs degradation

To be considered effective, it is evident that remediation strategies must be complemented by an efficient waste management. Recently, developing new methods to recycle plastic wastes by converting them into either their monomeric constituents or added-value products has become a fundamental target for realizing the circular economy concepts in the polymer life cycle.

Focusing on PET, 70 million tons of this plastic are annually produced worldwide [92], underlining the necessity of developing effective recycling approaches for this polymer. PET is made up of building block monomers, such as terephthalic acid (or benzene-1,4-dicarboxylic acid, H_2_BDC) and ethylene glycol, and it can be conveniently exploited as a source of organic ligands [93,94].

PET, like other thermoplastic polymers, such as PE, PP, PS and PC, can be recycled by melting through high temperature processes, and reintegrated into new products [95]. This recycling technology, however, consumes sizable amounts of energy and, upon melting, lower grade polymers are produced, with reduced thermal and mechanical stability, which limit the applicability of this technique to a reduced number of cycles [96]. The chemical degradation of PET and other plastics into their constituents represents another industrial viable approach [97]. Nevertheless, degradation through pyrolysis [96,98], in which plastics are processed at high temperatures in the presence of a catalyst, or catalytic cracking, which uses highly acidic zeolites, such as ZSM-5 and H-Y [99], also requires a considerable amount of energy or implies the emission of toxic gases, like halogenated acid or dioxins, which results into a negative environmental impact. Other chemical processes, including hydrolysis, methanolysis, glycolysis, aminolysis, and acid or base-catalysed hydrolysis [100–103] are employed for degradation of PET or other hydrolysable polymers, such as PLA and PU, into their starting monomers. These methods generally require the extensive use of solvents and other organic additives to promote degradation, which create secondary environmental issues that make these strategies not very sustainable [104]. Moreover, oligomeric side products or degraded dyes can also be afforded, which impact the polymer properties and make laborious separation processes necessary. Metal-based metal complexes have been recently studied as homogeneous catalysts, especially to catalyse the C–C bond cleavage in PP, LDPE and HDPE plastics [105,106]. These catalysts are often expensive, air-sensitive, feature a reduced selectivity towards low molecular weight degradation products, and their large-scale manufacturer is also far from being environmentally friendly.

Being plastic recycling principally motivated by economic factors, new less expensive and more stable catalyst materials are required. These catalysts must operate in relatively mild conditions, must be easily separated after use, and must display high turnover efficiency and elevated selectivity towards monomeric constituents.

It has been demonstrated that, if rationally designed, MOF-based heterogeneous catalysts and photocatalysts can feature outstanding catalytic activity and, in some cases, can outperform traditional homogeneous catalysts [107–109]. MOFs feature high active surface areas and stable porosity, with tuneable pore size and functionalities, which allow an easy microenvironment modulation. Their well-defined structures provide uniformly distributed catalytic sites in particular, Brønsted and Lewis acid sites at unsaturated metal centres, which contribute to improve the efficiency and selectivity of the catalytic process. Moreover, MOFs can afford more selective processes towards low molecular weight species, since their confined catalytic porous structure can hamper random-chain scission and recombination, thus controlling the molecular weight distribution of the degradation products. MOFs have been shown to catalyse a wide range of chemical reactions, including hydrolysis, oxidation, and olefin C–H activation [110–112]. MOFs have been also successfully employed for the degradation of different polymers, such as cellulose. For example, MIL-101(Cr)-SO_3_H was investigated as an efficient and clean catalyst for the hydrolysis/depolymerization of cellulose [113], due to the sulfonic groups decorating the pore surface, which acted as strong Brønsted acid sites. The bifunctional catalyst of Ru-PTA/MIL-100(Cr) was used to perform the one-pot conversion of cellulose into sorbitol with a high conversion rate [114]. Moreover, MOFs also showed enzymes-like activity for the hydrolysis reactions of different biopolymers, such as peptides and proteins or DNA molecules [115].

**Figure 4.**
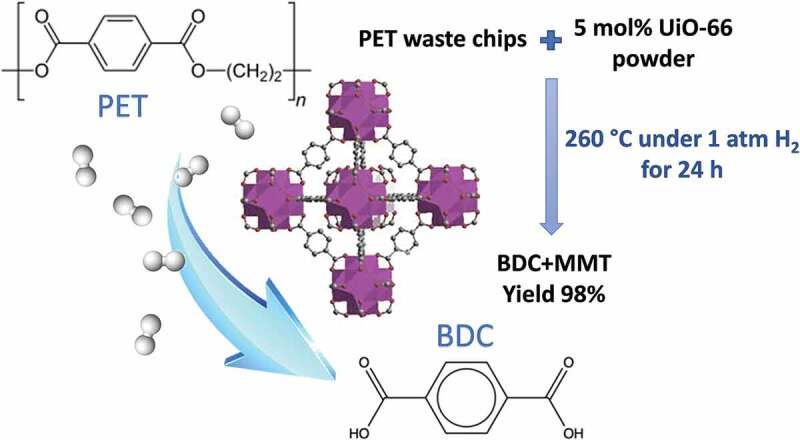
UiO-66 (Zr) decomposes PET waste into BDC and mono-methyl terephthalate (MMT) monomers under H_2_ atmosphere. Adapted from Ref. [48].

In a recent paper, UiO-66(Zr) MOF was reported to degrade waste PET into terephthalic acid and mono-methyl terephthalate in a solvent-free process within 24 h at 260°C (Figure 4) [48]. A conversion yield of 98% was obtained under 1 atm of H_2_, while the yield decreases to 81% under 1 atm of Ar. Notably, the effectiveness of UiO-66 catalyst, in terms of activity and selectivity for PET degradation, remains essentially unaltered even in the presence of PP or PE (molar ratio PET:(PP/PE) = 10: 1), with a conversion yields up to 93%. The PET degradation is accompanied by the structural transformation of the original UiO-66(Zr) MOF into a different Zr-based MOF, namely MIL-140A, that also demonstrated excellent catalytic properties for PET degradation under similar reaction conditions. These results demonstrated that MOFs can be profitably applied as a novel class of catalysts for MPs/NPs degradation and that they have the potential to tackle the issues related to plastic waste and its reuse.

## 4. Plastic waste as linker source for MOFs adsorbents

Several examples of PET-to-MOF conversion have been reported by different research groups [69–71].

Here, we provide a survey on the utilization of PET as a source of organic linkers for the sustainable preparation of some archetypal MOFs, while preserving the MOFs adsorption ability. This strategy could effectively address some issues related to the widescale preparation of MOFs, including high costs and not very viable synthesis routes, promoting at the same time their wider application.

### 4.1. MIL-47, MIL-53 and MIL-101

One of the early studies, which described the utilization of PET as a source of organic linkers for the synthesis of terephthalate-based materials was reported by Huang et al. in 2011, who described the preparation of a luminescent nanoporous zincophosphates [116]. Pursuing this purpose of using PET as the starting precursor for MOFs, an additional study appeared in 2016, describing the preparation of several different MOFs, i.e. MIL-47(V), MIL-53(Cr, Al, Ga) and MIL-101(Cr) [117]. Most of these MOFs were prepared in a one-pot reaction by solvothermal methods, in presence of HF or HCl to promote PET depolymerization. MIL-53(Cr) prepared according to this procedure was successfully used as adsorbing solid phase for the HPLC determination of various methylxanthines in tea samples [118]. The MOF-based matrix featured higher retention factors with respect to conventional silica-based columns, the enhancement being attributed to favourable π- π interactions between the alkaloid molecules and the aromatic rings of the BDC ligand.

**Figure 5.**
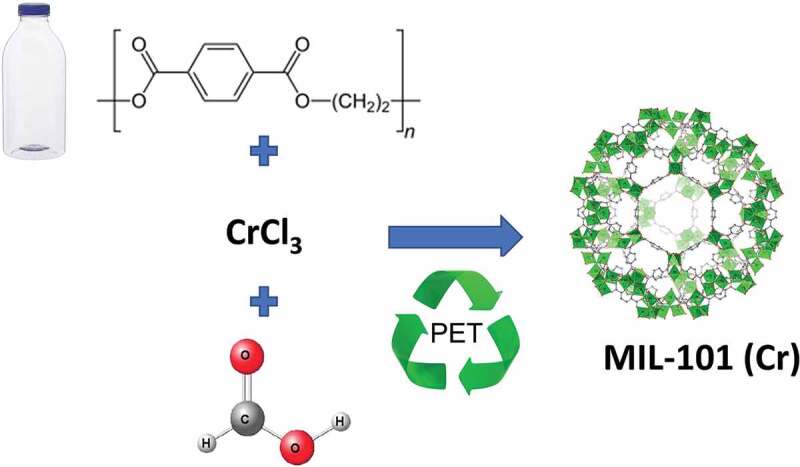
Direct synthesis of MIL-101 (Cr) from PET using formic acid as additive. Adapted from Ref. [119].

In the same year, Ren et al. utilized a greener approach for the preparation of MIL-101 (Cr) by using formic acid as additive instead of toxic HF (Figure 5) [119]. The reaction was carried out at 210°C for 8 h under autogenous pressure, with no need of purification steps. A PET-derived MOF-101 (Cr), produced following this procedure, was also functionalized through post-synthetic modification, and used for the effective and rapid removal of MB dye [120].

Pure phase MIL-53(Al) and MIL-47(V) were prepared by reacting PET with the appropriate metal salts in a microwave-assisted synthesis under hydrothermal conditions [121]. In this work, the authors demonstrated that PET hydrolysis and MOF synthesis can occur simultaneously. The use of metal chlorides increased the PET hydrolysis rate due to the *in-situ* generation of HCl from salt hydrolysis. Moreover, the hydrolysis process was further favoured by the MOF formation, since it sequestrates the produced H_2_BDC, thus preventing the precipitation of the ligand on the PET surface and the consequent interruption of the depolymerization reaction [122]. In the optimized conditions, MOF yield was 56%. They also showed that the PET bottle itself can be used as the synthesis reactor, when partially depolymerized, growing a MOF coating of MIL-53(Al) and UiO-66(Zr) on the PET substrate.

MIL-101(Cr) and MIL-101(Fe) were also prepared together with other MOFs, i.e. UiO-66(Zr) MIL-88B(Fe) and Al-Fumarate, following a two-step procedure, were the BDC linker was first prepared through the depolymerization of the PET debris with ethylene glycol, and then used for MOFs synthesis [123]. MOFs were tested for water adsorption processes, with MIL-101(Cr) exhibiting higher hydrothermal stability and adsorption properties.

### 4.2. UiO-66(zr) MOFs

Several examples of UiO-66(Zr) MOFs derived from PET have been also reported. Most of them describe the preparation of UiO-66(Zr) MOF by a two steps synthesis, where PET is first depolymerized and then the obtained BDC ligand is reacted with the opportune metal salt. For example, in 2016, Ren et al. reported the synthesis of UiO-66(Zr) from PET bottles [124]. PET was depolymerized in presence ethylene glycol under hydrothermal condition. The derived BDC ligand was then reacted with the zirconium chloride salt in DMF with the addition of formic acid. Moreover, by using coloured PET bottles in the process, the authors concluded that the additives and colourants have a minor effect on the final MOF properties. In a successive study, several kinds of PET wastes were selected and used as sources of BDC ligands for MOF UiO-66(Zr) preparation, including food trays and green and brown bottles [125], mainly demonstrating that the textural properties of the UiO-66(Zr) derived from waste PET were similar to those of the MOFs prepared with the commercial organic ligands. Nevertheless, in the case of the MOFs synthesized from coloured bottles, the organic dyes trapped within the MOF network was reported to reduced accessible porous areas.

PET-derived Zr-UiO(66) MOF, synthesized using BDC linkers obtained from PET bottles *via* alkaline hydrolysis, was used as adsorbent for the efficient removal of a nonsteroidal anti-inflammatory drug (NSAID), namely ketorolac tromethamine (KTC), from water media [126], the mechanism of adsorption being mainly determined by electrostatic interactions occurring between the MOF and the adsorbed molecules.

A one-step synthesis for the preparation of UiO-66(Zr) was described by Serre et al. [127]. The reaction was carried out at 160°C by tuning the reaction times, using acetone as solvent and in presence of formic acid. The organic acid worked as a modulator, leading to defects formation attributed to the partial lack of BDC ligands that further increased the final porosity of the MOF, while preserving the crystallinity. This PET-derived MOF was successfully used for the separation of benzene/cyclohexane, by exploiting the competing adsorption of these to molecules. Recently, the direct PET-to-UiO-66(Zr) MOF conversion in aqueous environment using different inorganic precursor and acids has been further investigated [128], proving that modified BDC ligands can be produced during the PET-to-MOF conversion. Indeed, the use of nitric acid for PET conversion resulted in the formation of NO_2_ groups on the aromatic rings of the BDC ligands, which were then reduced by the ethylene glycol produced by the PET depolymerisation, finally leading to the formation of NH_2_-UiO-66(Zr) MOF.

### 4.3. Other MOFs

Different research groups reported the preparation of other PET-derived-BDC MOFs containing Cu(II) [129–131], Zn(II) [132], Sn(II) [133], Ca(II) [134], Ba(II) [135], Ti(IV) [136], Co(II) and Ni(II) [137], as well as a lanthanide based (Tb) MOFs [138]. For all these MOFs, a two-step synthesis was proposed, where the BDC ligand was first produced from PET by either alkalosis, acidolysis, solvolysis or *via* a hydrothermal depolymerization with ethylene glycol. Most of these MOFs were applied either in adsorption, catalytic degradation, and sensing.

Recently, three different Fe-, La-, and Zr-MOFs were synthesized in a one-step reaction, where recycled PET bottles were directly employed as source of BDC ligands and applied for arsenate removal [139]. In particular, La-MOF exhibited remarkable adsorption capacity (114.28 mg/g at pH 7) for arsenate, performing better than Fe- and Zr-MOFs, as well as than other adsorbents reported in the literature.

A one-pot procedure for the large-scale preparation of Co-MOF nanorods from PET plastic waste was reported by Gong et al. [140]. A dual-functional Co-MOF/carbon nanotube membrane was also prepared and used as a sunlight driven evaporator and innovative photocatalyst for the oxidation of tetracycline in polluted water.

Mixed-ligand MOFs, namely [M_2_(BDC)_2_(DABCO)], where *M* = Fe(II), Co(II), Ni(II), Cu(II) and Zn(II); and DABCO = 1,4- diazabicyclo[2.2.2]octane), have been prepared in a one-pot reaction by employing PET waste as BDC source in presence of an inorganic acid to facilitate PET depolymerization [141], demonstrating that the topology of the PET-derived MOFs can be further tuned and expanded by adding other different organic ligands during the synthesis procedure.

## 5. Towards greener PET-to-MOF conversion

**Figure 6.**
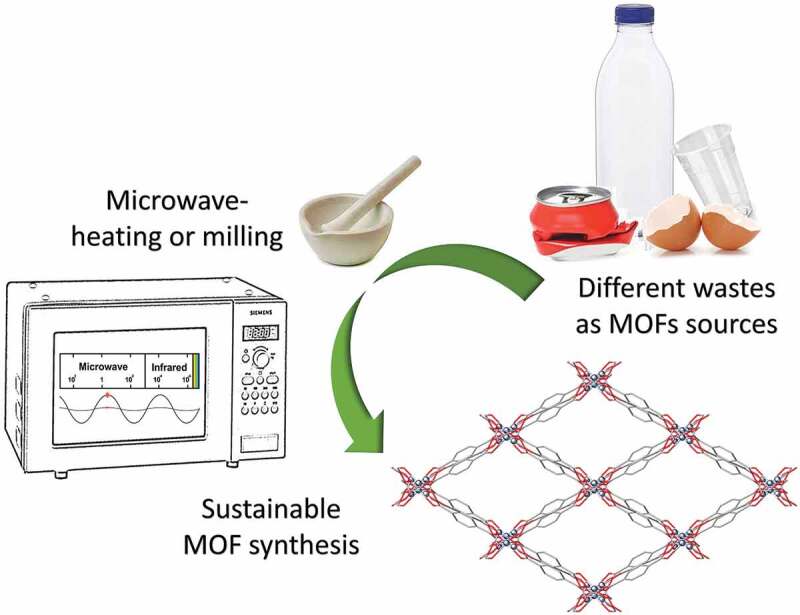
Sustainable sources and synthesis procedures for MOFs production.

Greener and more sustainable methodologies for MOFs syntheses also require reducing energy consumption, avoiding bulk solvent, or using water as reaction media, and implementing continuous manufacturing methods [62], see Figure 6. Microwave heating meets some of these requirements, since it provides reduced reaction times, faster crystal nucleation and growth, and higher yields and purity for the target product [142]. In 2013, Manju et al. introduced for the first time a two-steps PET-to-MOF conversion, where a microwave assisted alkalotic route was used for PET depolymerization [143]. This procedure allowed the preparation of high purity terephthalic acid, which was then used for the synthesis of two MOFs, MOF5 and [Cu(TPA)∙DMF]. The microwave assisted synthesis was also applied in the one-step PET-to-MOF conversion proposed by Deleu in 2016 [121] which was discussed above. Recently, it was also employed to prepare an Al-MOF, which was used as effective adsorbent for the removal Eriochrome Black T (EBT) dye, a contaminant for synthetic wastewater [144].

One of the most recent works on the use of PET as ligand source reported a mechanochemical milling strategy to convert PET into different MOFs, containing La(III), Zr(IV), Ni(II), Co(II), Mn(II) and Ca(II) [145]. By mixing PET and NaOH, PET was first hydrolysed to Na_2_BDC. Then, the ligand was mechanochemically reacted with the opportune metal salt. This promising strategy has several benefits, including solvent-free conditions, use of ambient temperature, reduced reaction time, and straightforward scale-up for large-scale MOFs synthesis.

Finally, some examples of MOFs entirely derived from waste, i.e. prepared by using different environmental-friendly or recycled materials as sources for both metal ions and organic linkers, have been reported. MIL-53(Al) was synthesized from aluminium foil/can as Al(III) source and PET bottles as precursor for BDC linkers [146]. In this case, aluminium nitrate and BDC linkers were first derived from the corresponding waste source and then used for the MOF synthesis. Ca(BDC)(H_2_O)_3_ was synthesized from eggshells as source of Ca(II) ions and recycled PET bottles as precursors for the BDC ligands. The one-pot solvothermal synthesis was carried out by directly reacting a mixture of crushed eggshells and PET pieces in water and ethylene glycol [147].

## 6. Conclusions, challenges, and perspectives

Recent developments in MOFs applications suggest that MOFs can be effectively applied for MPs/NPs adsorption or catalytic degradation from water, opening a promising route for the simultaneous removal of NPs and other dissolved micropollutants eventually present in complex matrices. To tackle the challenges linked to the direct use of MOFs as nanocrystalline powders, MOFs can be conveniently shaped as bulk materials. Another possible viable strategy involves the design of more complex MOF-based heterostructures. Recently, nanopillared structures composed of 2D MOF intercalating carbon encapsulating iron oxide nanoparticles (C@FeO) have been proposed for the simultaneous removal of both MPs and MB molecules [148]. PS beads (mean diameter of 1 μm) were chosen as model MPs. In the optimized experimental condition, the 2D MOF@C@FeO removed up to 100% of MPs in 60 min. Furthermore, they also removed both MP and MB molecules in 60 min. The stability of the 2D MOF@C@FeO structure and its reusability was proved in 6 absorption cycles with 90% removal capacity. The C@FeO particles introduce useful magnetic properties which can help to separate MOF from solution after adsorption, in an easier way if compared to filtration or centrifugation.

MOF-based membranes have the potential to effectively remove MPs and, above all, the more challenging NPs, as well as other coexisting microcontaminants from wastewater in a single process, while maintaining efficient water permeability and antifouling properties. By exploiting a combination of size exclusion and adsorption through week interactions (i.e. hydrogen bonding, π-π stacking, etc.), MOF-based membranes can retains ions, molecules and nanosized pollutants, helping to overcome some intrinsic limitations of conventional polymeric membranes, linked to the low particle size that NPs can reach. Unfortunately, few examples are reported in literature regarding the use of MOFs and MOF-based composite materials for MPs/NPs removal. This field deserve to be further investigated, since it has the potential to broaden the application of MOFs and MOFs-based materials for water remediation. One of the major challenges in MOF-based membranes can be maintaining the required performance in complex wastewater matrices with diversified contaminants. Future studies should focus on the design of tuneable MOFs with different framework structures, surface charges and functional groups, in order to target diverse micropollutants.

With respect to their use as catalysts for MPs and NPs degradation, MOFs are expected to show enhanced catalytic activity for the degradation of polyester-based plastic such as PET, as confirmed by the early study reported in literature. Nevertheless, MOFs have been also successfully employed for the degradation of other different polymers. Examples includes the C-O bond cleavage of cellulose, and the hydrolysis of peptide bonds or the phosphodiester linkages in biopolymers [113–115]. Moreover, Ru@ZIF-8 catalyst with extremely low metal Ru loading has been recently used for the selective hydrogenolysis of C–O bonds in lignin model compounds [149], and the aerobic C–C bond cleavage of a lignin model compound was also reported using a cationic MOF of formula [Cu_2_(L)(H_2_O)_2_](NO_3_)_2_·5.5 H_2_O (L = 1,1′-bis(3,5-dicarboxylatophenyl)-4,4′-bipyridinium), whose activity resulted to be dependent from anions included within the porous structure [150]. All these findings have the potential to broaden the application of MOF catalysts even for the degradation of more recalcitrant plastics.

Finally, designing MOFs with mixed linkers or mixed metal centres can also offer an unprecedented opportunity to construct multifunctional active sites for multiple, synergic or cooperative catalytic reactions [151,152].

Future studies on MPs/NPs removal should be also profitably extended to Covalent Organic Frameworks (COFs), a class of crystalline porous organic materials made up of organic building blocks connected through robust covalent bonds [153]. Like MOFs, COFs feature high chemical stability, regular porous networks, large surface area; and versatile structures, which allow easy surface modification. They can also be usefully integrated in functional composite materials [154] or processed to form hierarchically porous adsorbents [155]. COFs have been investigated for different applications, including adsorption of different pollutants from water and catalytic, electrocatalytic and photocatalytic applications for several reactions, demonstrating enhanced performances and good recyclability [156,157]. Despite the adsorption of polymers on COFs has been scarcely investigated, molecular dynamics simulations provided some promising results, revealing that strong interfacial adsorption of PE, PET and nylon-6 (PA 6) NPs can occur with some stable COFs (TpPa-X, X = H, CH3, OH, NO2 and F) [158]. Additionally, COFs also demonstrated suitable catalytic activities. For example, the selective activation of various C-H bonds, and recently, also the aerobic oxidative C-C bond cleavage of cycloalkanones has been reported for metal-free quinoline COF-based photocatalyst, decorated with Brønsted acid sites [159,160].

The high cost that could derive from the inclusion of MOFs as adsorbent fillers in membranes or other composite MOF-based materials can be mitigated by the implementation of new synthetic routes, which can encompass the use of cheaper chemicals and procedures. The use of recycled wastes as source for both metals and organic ligands could help to partially solve this issue, also contributing to the implementation of greener methods for MOF production. The PET-derived MOFs demonstrated good performances when used as adsorbents for different pollutants, comparable to the MOFs prepared with commercial and expensive organics ligands.

Even if many plastics produced at a large scale, i. e. polyethylene, polypropylene, and polybutylene, are not appropriate as source of linkers for MOF preparation, since the correspondent constituents do not possess suitable functional groups for metal coordination, additional plastic wastes can be investigated as potential sources for organic ligands. For example, polybutylene terephthalate (PBT), largely employed in electrical and insulating items, can be another suited source of BDC ligand. Some polyoxometalate MOF (POMOF) have been synthesized through a one-step procedure using either PET or PBT as BDC source, with a conversion close to 100% [161]. Lactic acid-based MOFs (LA-MOFs) can in principle be synthesized by employing recycled polylactic acid (PLA) as a source for the organic ligand. Ladewig et al. first described the use of waste PLA cups as a precursor for LA linkers. They have recently synthesized three homochiral MOFs with a one-step procedure, exploiting the *in situ* depolymerized of PLA [162]. In a typical synthesis, ZnBLD MOF was prepared by reacting zinc nitrate salt, terephthalic acid and PLA cups in DMF at a temperature of 110°C for 48 h.

One of the most serious drawbacks of MOFs derived from recycled waste is the potential reduction of their active porous area caused by the inclusion of impurities or the formation of defects in the crystalline structure. With this respect, the use of PET waste for BDC-based MOFs requires further investigation for significantly improving PET conversion and MOFs purity in order to achieve the industrial feasibility.
